# Modeling and Simulating Passenger Behavior for a Station Closure in a Rail Transit Network

**DOI:** 10.1371/journal.pone.0167126

**Published:** 2016-12-09

**Authors:** Haodong Yin, Baoming Han, Dewei Li, Jianjun Wu, Huijun Sun

**Affiliations:** 1 State Key Lab of Rail Traffic Control & Safety, Beijing Jiaotong University, Haidian District, Beijing, P.R. China; 2 School of Traffic and Transportation, Beijing Jiaotong University, Haidian District, Beijing, P.R. China; West Virginia University, UNITED STATES

## Abstract

A station closure is an abnormal operational situation in which the entrances or exits of a rail transit station have to be closed for some time due to an unexpected incident. A novel approach is developed to estimate the impacts of the alternative station closure scenarios on both passenger behavioral choices at the individual level and passenger demand at the disaggregate level in a rail transit network. Therefore, the contributions of this study are two-fold: (1) A basic passenger behavior optimization model is mathematically constructed based on 0–1 integer programming to describe passengers’ responses to alternative origin station closure scenarios and destination station closure scenarios; this model also considers the availability of multi-mode transportation and the uncertain duration of the station closure; (2) An integrated solution algorithm based on the passenger simulation is developed to solve the proposed model and to estimate the effects of a station closure on passenger demand in a rail transit network. Furthermore, 13 groups of numerical experiments based on the Beijing rail transit network are performed as case studies with 2,074,267 records of smart card data. The comparisons of the model outputs and the manual survey show that the accuracy of our proposed behavior optimization model is approximately 80%. The results also show that our model can be used to capture the passenger behavior and to quantitatively estimate the effects of alternative closure scenarios on passenger flow demand for the rail transit network. Moreover, the closure duration and its overestimation greatly influence the individual behavioral choices of the affected passengers and the passenger demand. Furthermore, if the rail transit operator can more accurately estimate the closure duration (namely, as *g* approaches 1), the impact of the closure can be somewhat mitigated.

## Introduction

Station closure, which is also called station disruption [1], is an abnormal and unplanned operational situation in which operators must close the entrances or exits of a rail station for various reasons, such as unexpected incidents or taking steps to avoid overcrowding. In these situations, passengers cannot use the closed station as their departure station or destination station for some time. Generally, station closure is a serious deviation from the planned operations in the rail transit context. A station closure can strongly affect both the service and demand of the rail transit system. At the service level, a station closure means that the trains that were planning to stop at the closed station have to pass the closed station without stopping. At the demand level, the station closure may cause significant changes in passenger flow demand at the closed stations and nearby stations. The affected passengers may react to the station closure in different ways. They may alter their planned origin station or destination station, wait to continue on their original path, reroute through the rail transit network, or simply give up on the rail transit journey.

However, limited existing studies are available related to the qualitative analysis or modeling of passengers’ reactions to segment disruptions or line disruptions [1–8]. To our knowledge, only one study has been conducted related to the topic of station disruption [1]. In that study, a data-driven statistical method was proposed to determine the effect of station disruptions on the macroscopic passenger flow demand based on the smart card data from the day of the station disruption and the historical data. This approach was proved to be effective for identifying the impact of a station closure, but it is not applicable for predicting the impact of a station closure that does not actually occur or is about to occur. Therefore, an effective mathematical model or simulation model that can capture the behavior of passengers affected by a station closure is still needed for analyzing alternative closure scenarios and their likely outcomes.

In this paper, we develop a novel approach for estimating the impact of a station closure in the rail transit network on both the passenger behavioral choices at the individual level and passenger demand at the disaggregate level. In addition, we assume that the demand is the result of several decisions made by each individual in the population. Therefore, the individual behavior model is the basis for estimating the passenger demand. The contributions of our work are two-fold: First, a basic passenger behavior optimization model is mathematically constructed based on 0–1 integer programming to describe passengers’ responses to alternative origin station closure scenarios and destination station closure scenarios; this model also considers the availability of multi-mode transportation and the uncertain duration of the station closure. Second, an integrated solution algorithm based on the passenger simulation is developed to solve the proposed model and to estimate the effects of station closure on passenger demand in the rail transit network.

The paper is organized as follows: the next section gives a detailed review of related studies. In the section of Methods, the detailed problem description and the passenger behavior model framework are introduced. Then, a passenger behavior optimization model that considers the dynamics and uncertainty of the closure duration is proposed for the case of a station closure. Then, an integrated solution algorithm based on the passenger flow simulation is developed to solve the model. The case study results are reported in the Case study section. Finally, some conclusions are given in the section of Conclusions.

## Literature Review

Existing approaches for studying the effects of disruptions on traffic behavior can be categorized as data-driven methods or model-driven methods. ***The data-driven method*** is used to recognize patterns of passenger behavior or to directly evaluate or predict the traffic conditions based on the data [1, 6, 9–10]. In the data-driven methods, huge amounts of operational data are processed, and then statistical models or demand forecasting models are developed. ***The model-driven method*** is used in cases that lack sufficient real-life data. A qualitative analysis of the behavior mechanism and the main behavioral decision factors are first needed; then, the simulation models or the analytical models are constructed. Finally, the models are tested based on real-life data to capture the passenger/driver/cyclist behavior and to estimate the dynamic evolution of macro traffic flow under disruptions [11–20]. Therefore, the detailed literature review mainly focuses on these two methods.

### (1) The data-driven methods

With the development of Automatic Fare Collection Systems [10, 21], Vehicular Ad Hoc Networks [21–27], and the latest Cyber-Physical Systems [28], huge traffic data has been widely used in recent transportation studies and practical applications. In the urban rail transit context, Silva *et al*. [1] modeled passenger behavior under two types of disruptions: bidirectional line segment closures and station closures. They proposed a statistical method using network-wide smart card data to estimate the effects of disruptions due to unplanned station or line closures. van der Hurk *et al*. [6] presented a two-step methodology for studying the effect of large-scale disruptions on passenger behavior based on smart card data. The methodology was able to forecast the passengers affected by a disruption. However, this methodology assumed that passengers only altered their path choice and did not choose a different mode of transport. Bouman *et al*. [9] analyzed passenger demand patterns from smart card data. They combined three types of demand that can be detected in a smart card dataset, i.e., trip-based demand, tour-based demand and pattern-based demand, which form the basis of generating agent populations. Kusakabe T *et al*. [10] developed a data fusion methodology for estimating the behavioral attributes of trips using smart card data to observe continuous long-term changes in trip attributes.

### (2) The model-driven methods

The model-driven methods can be categorized into analytical models and simulation models according to the nature of their modeling and solution approaches. The analytical models are mainly mathematical optimization problems with a specific objective (such as User Equilibrium (UE) or System Optimal (SO)) [12–13] or discrete choices models. In the urban rail transit context, Nuzzolo *et al*. [14] proposed a schedule-based assignment model that defined path choice behavior in two parts: (1) the pre-trip choice behavior, which includes the departure time and the boarding station, and (2) the en-path choice behavior, which mainly includes the decision to board an arriving train considering its residual capacity. Then, they designed a learning mechanism based on the dynamic utility of three choice dimensions: run, stop and departure times. However, the disruption factors were not considered. Cadarso *et al*. [4, 5] used a general multinomial logit model to describe the general process of passenger travel decision making when facing segment disruptions. The utility function of each path is calculated as the sum of the traveling time, the transfer time and the waiting time. In addition, the travel time is estimated under disruptions before calculating the utility function, as in Tsuchiya *et al*. [7]. Bouman *et al*. [8] focused on passenger path choice behavior for the case of track disruptions, where a passenger has to decide between waiting for the end of the disruption and taking a detour. Then, they gave an approach based on game theory and adopted the competitive ratio in the decision process. Behavior simulation models are a popular tool in assessing the traffic system performance and analyzing the behavior of passengers in alternative scenarios. The passenger behavior simulation models can also generally be categorized into microscopic, macroscopic or mesoscopic according to the granularity of the modeled traffic flow entities and the temporal resolution [11]. The mesoscopic simulation model is mostly used to describe the passenger behavior for alternative scenarios. Cats [16–18] established an event-based passenger behavior simulation model on the mesoscopic level, in which each action, such as entering a station and boarding/alighting a train, was an event. Othman *et al*. [19] developed an agent-based simulation model of congestion and scaling dynamics of rapid transit systems in the regular situation. The agent-based methods were also used for modeling microscopic pedestrian behavior [20].

In summary, we can see that few studies discuss the topic of station closure. In addition, the only related study proposed a data-driven statistical method for recognizing the effects of station disruptions that have already occurred based on historical smart card data. Making a predictive assessment of the impact of alternative station closure scenarios that do not actually occur or are about to occur remains an unsolved problem. Therefore, in the next section, a new model-driven method for modeling the passenger behavior under alternative station closure scenarios is proposed.

## Methods

The smart card data, which were used to generate passenger individuals, were analyzed anonymously. This data is introduced in the next section in detail.

In this section, we first give a detailed description of the passenger behavior modeling problem with station closures. Then, a dynamic passenger behavior model for the case of station closures is formulated, and an integrated solution algorithm based on the passenger flow simulation is developed.

### Problem description and modeling framework

**Fig 1:**
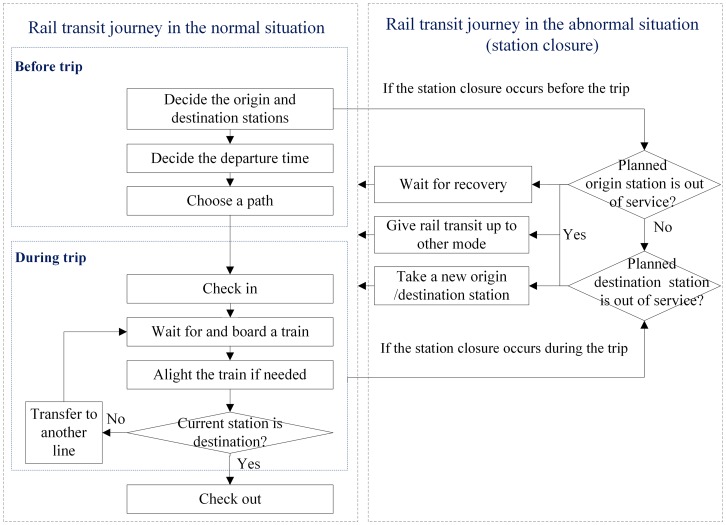
A schematic diagram indicating the passengers’ travel behavior along a rail transit journey in the normal and abnormal conditions. A detailed description is presented in the previous paragraph and the next paragraph. In addition, this logic diagram is a qualitative summary of passenger travel behavior in a rail transit network and is originally presented in this paper.

Large differences in passenger behavior may occur between the normal and abnormal operational situations, as shown in Fig 1. In the normal situation, passengers complete a rail transit trip in two stages [14]. The first stage is the pre-trip choice behavior that occurs before the trip and includes deciding on an origin station, destination station and departure time and choosing a path from all feasible candidate paths. The second stage is the en-path behavior that occurs during the trip and includes checking in at the origin rail station, waiting for a train on the platform, boarding a train, and then alighting the train at the destination station or transfer station. If the current station is the passenger’s destination, the passenger will check out; if the current station is simply a transfer station, the passenger will transfer to another line.

However, in the abnormal situation, the passengers affected by the disruption may change their travel behavior, as shown in Fig 1. If the abnormal incident occurs before the trip, the passengers will make a judgment about whether their planned origin or destination stations are out of service, and the affected passengers may modify their trip plans or make a new trip decision. However, if the closure occurs during their trips, the passengers will judge whether their planned destination stations are out of service and plan an alternative trip from the current station to the new destination.

**Fig 2:**
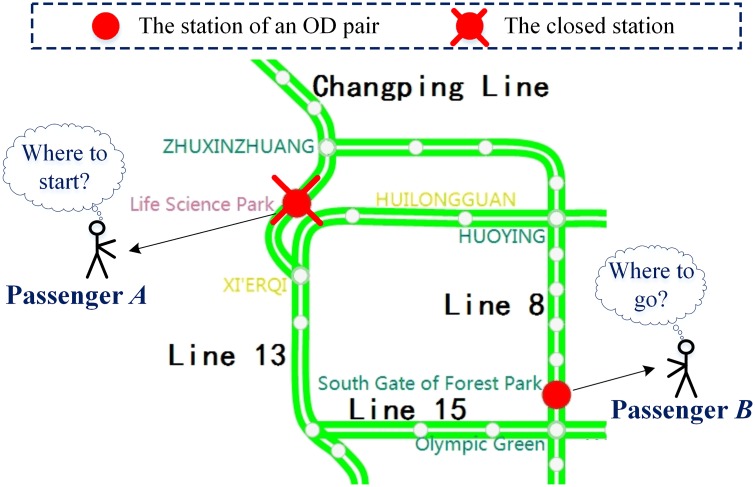
An example of a station closure in the Beijing rail transit network. As indicated in this example, the Life Science Park station on the Changing Line is out of service. Passenger *A* is going to depart from this station by rail transit. Passenger *B* is currently at the South Gate of Forest Park station on Line 8, and his destination is the Life Science Park station.

Many abnormal incidents, such as injury accidents at a station or taking steps to avoid overcrowding due to a large event nearby, can lead to the closure of a rail transit station. In such situation, passengers cannot board or alight trains, and trains pass the station without stopping, as mentioned by Silva *et al*. [1]. When a station closure occurs, the affected passengers face two possible situations: the closed station is their planned origin station or the closed station is their planned destination station. As shown in Fig 2, passenger *A*, whose departure station is out of service, may change their trip plan according to information about the current situation such as the closure duration and the availability of alternative paths. They may also choose an alternative station near the closed station as their new departure station, wait at the closed station for recovery, or take another transit mode. Passenger *B*, whose destination is out of service, has to decide whether to choose an alternative destination station or take another transit mode.

Therefore, understanding passenger behavior in the case of a disruption is important. When an origin or destination station is out of service in a rail transit system, the affected passengers will decide on an alternate station as their new origin or destination station. However, which rail station is the best alternative among the stations in the network? In addition, how does the passenger get to the new origin rail station from the disrupted origin station or to the disrupted destination station from the new destination station? It is difficult to accurately capture the behavior under the station closure condition. Therefore, in this paper, we propose an agent-based behavior optimization model to discover the mechanism of the dynamic behavior subject to alternative station closure scenarios. To solve the proposed model, an integrated solution algorithm embedded in a passenger simulator is developed.

### Assumptions

To simplify the problem, the following assumptions are made.

**Fig 3:**
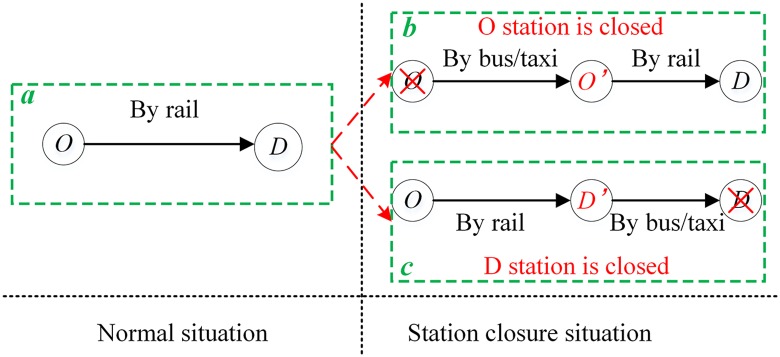
An illustration of assumption 2. For a particular rail passenger, O and D denote the planed origin and destination stations, respectively. *O’* denotes the alternative origin station when the planed origin station is closed, and *D’* denotes the alternative destination station when the planed destination station is closed. (*a*) represents the passenger’s planned rail journey in the normal situation. (*b*) shows that the passenger will take a bus or taxi to the new alternative origin station and will then take rail transit from the new origin station to the planned destination when the planned origin station is closed. (*c*) shows that the passenger will take rail transit from the planned origin station to the new alternative destination and will then take a bus or taxi to the planned destination station then when the planned origin station is closed. Only the two situations of (*b*) and (*c*) are considered in our studies for station closure.

(1) The duration of the station closure can be estimated. In reality, not even the operator knows the actual ending time of the station closure [8]. However, the operators will be able to estimate the duration of the closure based on their experience. Only in this way can they make arrangements for measures or resources to address the coming closure and disruption. But, the estimates are usually more or less in line with actual values. Therefore, a parameter related to the closure duration is proposed in our study.

(2) Passengers can transform their whole trip into a maximum of two combinations when subject to a station closure, as shown in Fig 3. Specifically, a planned rail trip (shown in Fig 3(a)) can only be re-planned as a combination of “by bus/taxi” and “by rail” in sequence following the closure of the origin station (shown in Fig 3(b)) or as a combination of “by rail” and “by bus/taxi” in sequence following the closure of the destination station (shown in Fig 3(c)). The combination of three or more parts, such as “rail”, “bus/taxi” and “rail” in sequence, is not used in our studies.

(3) We limit our study to the scenario in which only the origin station or the destination station is closed in the particular time interval for a particular passenger. We do not consider the possibility that both the origin station and destination station are closed simultaneously for a passenger. In addition, the closed station is assumed to be an ordinary station, not a transfer station. The closure of a transfer station may cause a failure to transfer, which is not considered in our behavior model.

### Model formulations

#### Notations

**(1) Sets and indices**

*S*: set of stations, *S* = {*s*_1_, *s*_2_,…,*s*_*n*_}. Here, the transfer station is abstracted as two or three virtualized stations, the number of which equals to the number of lines that the transfer station links; see Ref. [29].

*T*: set of equivalent time intervals, *T* = {1, 2, 3,…,*T*_*n*_}. The time interval can be any value such as 30 seconds, 1 minute, or 5 minutes.

*t*: index of the equivalent time intervals for passengers to make a new travel decision for the case of a station closure, *t* ∈ *T*.

*P*: set of passengers in the current rail transit system.

*M*: set of modes of urban transport, *M* = {*taxi*, *bus*, *rail*}.

*M*′: subset of *M*, *M*′ = {*taxi*, *bus*}.

*S*_*i*_, *s*_*j*_: indexes of stations, *s*_*i*_,*s*_*j*_ ∈ *S*.

*m*: index of the urban transport mode in *M*, *m* ∈ *M*.

$P_{ij}^{t}$: set of passengers from station *s*_*i*_ to station *s*_*j*_ at time *t*, *$P_{ij}^{t}\subset P$*.

$R_{ij}^{m,t}$: set of candidate paths of urban transport mode *m* from station *s*_*i*_ to station *s*_*j*_ at time *t*. The candidate paths of the rail transit network are calculated by our proposed method [30], and the other paths refer to the *Direction API* of Baidu map [31].

$p_{ij}^{t}$: index of the passenger with the origin station *s*_*i*_, destination station *s*_*j*_, and departure time of time *t*, $p_{ij}^{t}\in P_{ij}^{t}$.

$r_{ij}^{m,k,t}$: the *k*th candidate path of urban transport mode *m* from station *s*_*i*_ to station *s*_*j*_ at time *t*, *k* ≤ *K*. In addition, *K* is a constant denoting the maximum number of candidate paths.

**(2) Passenger travel strategy parameters**

*α*^*m*^: the value of the travel time of urban transport mode *m*, which can be found in local studies in China [32,33].

$t_{ij}^{m,t,k}$: the travel time for taking transport mode *m* along the *k*th path from station *s*_*i*_ to station *s*_*j*_ at time *t* in the normal situation.

$f_{ij}^{m,k,t}$: the fee for taking transport mode *m* along the *k*th path from station *s*_*i*_ to station *s*_*j*_ at time *t*.

$c_{ij}^{m,k,t}$: the generalized cost of taking transport mode *m* along the *k*th path between station *s*_*i*_ and station *s*_*j*_ at time *t* considering the travel time and price.

**(3) Station closure parameters**

$\gamma_{i}^{t}$: the constant parameter denoting whether station *s*_*i*_ is closed. $\gamma_{i}^{t}$ equals 1 when station *s*_*i*_ is out of service and is 0 otherwise.

$t_{i}^{start}$: the beginning time of the closure of station *s*_*i*_.

$t_{i}^{end}$: the actual ending time of the closure of station *s*_*i*_.

*g*: a constant coefficient related to the actual duration, *g* ∈ [1, ∞), indicating the operator’s decision preferences. A larger *g* is produced by a more conservative operator. *g* = 1 indicates that the operator knows the exact ending time of the station closure. g = ∞ indicates that the operator has no idea how long the station closure will last and that it will perhaps last until the end of daily operations.

$e_{ij}^{m,k,t}$: the extra journey time cost of rail transit along the *k*th path from station *s*_*i*_ to station *s*_*j*_ at time *t* due to the station closure:

$$e_{ij}^{m,k,t}=\{\begin{array}{ll} g*(t_{i}^{end}-t), & \text{if }s_{i}\text{ is closed and }m=rail,\;t_{j}^{end}>t\\ g*(t_{j}^{end}-t-t_{ij}^{m,k,t}), & \text{if }s_{j}\text{ is closed and }m=rail,\;t_{j}^{end}>t\\ \text{0,} & \text{otherwise} \end{array}$$ (1)

#### Model formulation

First, we use *s*_*O*_ and *s*_*D*_ to denote the planned origin station and the destination station, respectively, of the passenger’s rail transit journey, where *s*_*O*_, *s*_*D*_ ∈ *S*. For the passenger $p_{OD}^{t}$, we construct a behavior optimization model to describe their decision-making process for the case of station closure.

**(1) Decision variables**

$x_{i}^{t}$: the main decision variable denotes whether station *s*_*i*_ should be chosen as the alternative origin or destination station at time *t*, where

$$x_{i}^{t}=\{\begin{array}{ll} 1, & \text{if station }s_{i}\text{ is selected as the replaceable origin/destination station;}\\ 0, & \text{otherwise.} \end{array}$$ (2)

$z_{ij}^{m,t}$: the decision variable denotes whether the transport mode *m* should be the alternative transport mode from station *s*_*i*_ to station *s*_*j*_ at time *t*, where

$$z_{ij}^{m,t}=\{\begin{array}{ll} 1, & \text{if tranport mode }m\text{ is selected as the replaceble transport mode between }si\text{ and }sj\text{;}\\ 0, & \text{otherwise.} \end{array}$$ (3)

$y_{ij}^{m,k,t}$: the decision variable indicates whether the *k*th path should be chosen from the feasible path set between station *s*_*i*_ and station *s*_*j*_ at time *t* while taking transport mode *m*.

$$y_{ij}^{m,k,t}=\{\begin{array}{ll} 1, & \text{if }k\text{th route of transport mode }m\text{ is eventually selected between }si\text{ and }sj\text{;}\\ 0, & \text{otherwise.} \end{array}$$ (4)

**(2) Objective and constraints**

For any passenger $p_{O,D}^{t}$, the objective of this behavior model is to minimize the generalized travel cost of making a new travel strategy for the case of a station closure.

Objective

$$\text{min}\sum_{j\in \{O,D\}}\gamma_{j}^{t}J_{j}^{t}$$ (5)

Subject to

$$J_{O}^{t}=\sum_{s_{i}\in S}x_{i}^{t}(\sum_{m\in M^{\prime }}\left(z_{O,i}^{m,t}\sum_{k}^{K}(y_{O,i}^{m,k,t}c_{O,i}^{m,k,t}\right))+\sum_{k}^{K}(y_{i,D}^{rail,k,t}c_{i,D}^{rail,k,t})),\;\forall t,p_{O,D}^{t},O\text{ is out of service}$$ (6)

$$J_{D}^{t}=\sum_{s_{i}\in S}x_{i}^{t}(\sum_{k}^{K}(y_{O,i}^{rail,k,t}c_{O,i}^{rail,k,t})+\sum_{m\in M^{\prime }}\left(z_{i,D}^{m,t}\sum_{k}^{K}(y_{i,D}^{m,k,t}c_{i,D}^{m,k,t}\right))),\;\forall t,p_{O,D}^{t},D\text{ is out of service}$$ (7)

$$\gamma_{O}^{t}+\gamma_{D}^{t}=1,\;\forall t,p_{O,D}^{t}$$ (8)

$$\sum_{s_{i}\in S}x_{i}^{t}=1,\forall t$$ (9)

$$\sum_{m\in M^{\prime }}z_{ij}^{m,t}\leq 1,\forall t,i,j$$ (10)

$$\sum_{k}^{K}y_{ij}^{m,k,t}\leq 1,\forall t,m,i,j$$ (11)

$$c_{ij}^{m,k,t}=\alpha^{m}*(t_{ij}^{m,k,t}+e_{ij}^{m,k,t})+f_{ij}^{m,k,t},\forall t,m,k,i,j$$ (12)

$$t_{i}^{start}\leq t\leq t_{i}^{end},\forall i=O\;or\;D$$ (13)

Objective function (5) minimizes the generalized travel cost. It is the combination of two situations: the closure of the origin station and the closure of the destination station. However, for a particular passenger $p_{O,D}^{t}$, only one situation satisfies constraints (6)–(8).

Eqs (6)–(8) indicate the basic constraints of the station closure situation for a particular passenger. Constraint (6) denotes the generalized travel cost of making a new journey plan if the passenger’s origin station is out of service. Constraint (7) denotes the generalized travel cost of making a new journey plan if the passenger’s destination station is out of service. Constraint (8) indicates that only one case occurs for a particular passenger, which means that either the origin station ($\gamma_{O}^{t}=1,\;\gamma_{D}^{t}=0$) or the destination station ($\gamma_{O}^{t}=0,\;\gamma_{D}^{t}=1$) is closed. Other cases, such as the cases of $\gamma_{O}^{t}=1,\;\gamma_{D}^{t}=1$ or $\gamma_{O}^{t}=0,\;\gamma_{D}^{t}=0$, are beyond the scope of this model.

Eqs (9)–(11) indicate the constraints of the decision variables. Constraint (9) indicates that an alternative origin or destination station will be chosen. Constraint (10) donates that one alternative transport mode will bridge the invalid original origin station to the new alternative origin station or the new alternative destination station to the invalid original destination station instead of the rail transit mode. In addition, if *i* = *j*, $\sum_{m\in M^{\prime }}z_{ij}^{m,t}=0$. Constraint (11) donates that a feasible path will be chosen from the optional paths for a particular transport mode m between station *s*_*i*_ and station *s*_*j*_. Finally, if *i* = *j*, $\sum_{k}^{K}y_{ij}^{m,k,t}=0$.

Eq (12) shows how to calculate the generalized cost of taking transport mode *m* along the *k*th path between station *s*_*i*_ and station *s*_*j*_ at time *t* considering the travel time and price. As defined in Eq (1), this considers a dynamic time variable that will change over time.

Eq (13) indicates that the time interval for making a decision for a particular passenger occurs during the period of the station closure. Specifically, the model is applicable when the passenger has just been faced with the station closure.

#### Output analysis of the behavior model

This model addresses a particular passenger’s travel decision problem when faced with the closure of the origin station or destination station. As our decision variables are 0–1 integer variables, and the model objective is to minimize the value of the objective function, our proposed passenger behavior model is belonged to optimization models based on 0–1 integer programming. That also means the outputs of our proposed behavior model are optimal for the passengers under the effect of alternative station closure scenarios.

(1) The model outputs under origin station closure ($\gamma_{O}^{t}=1$): This case occurs before the passenger starts his rail transit journey. If $x_{i}^{t}=1$ and *i* = *O*, the passenger will not change his travel strategy but instead wait for the recovery of his original station. If $x_{i}^{t}=1$ and *i* = *D*, the passenger will give up on the rail transit portion of their journey and will take a bus or taxi instead. If $x_{i}^{t}=1$ and *i* ≠ *O*, *D*, the passenger should take a bus or taxi from the old origin station *s*_*O*_ to the new origin station *s*_*i*_ and then continue their rail transit journey from the new origin station *s*_*i*_ to the destination station *s*_*D*_.

(2) The model outputs under destination station closure ($\gamma_{D}^{t}=1$): This case may occur before the passenger starts their rail transit journey or during their rail trip. In addition, if the passenger is on a rail transit trip, such as when they are at a transfer station or on board a train, the current station will be processed as the origin station in our model. Then, the passenger will make a decision about whether to change their travel strategy. For the situation of ($\gamma_{D}^{t}=1$), the following options are possible: If $x_{i}^{t}=1$ and *i* = *O*, the passenger will give up on the rail transit portion of the journey and take a bus or taxi instead. If $x_{i}^{t}=1$ and *i* = *D*, the passenger will not change their travel strategy. If $x_{i}^{t}=1$ and *i* ≠ *O*,*D*, the passenger should take the rail transit from the origin station *s*_*O*_ to the new destination station *s*_*i*_ and continue their journey by bus or taxi from the new destination station *s*_*i*_ to the original destination station *s*_*D*_.

### An integrated solution algorithm

**Fig 4:**
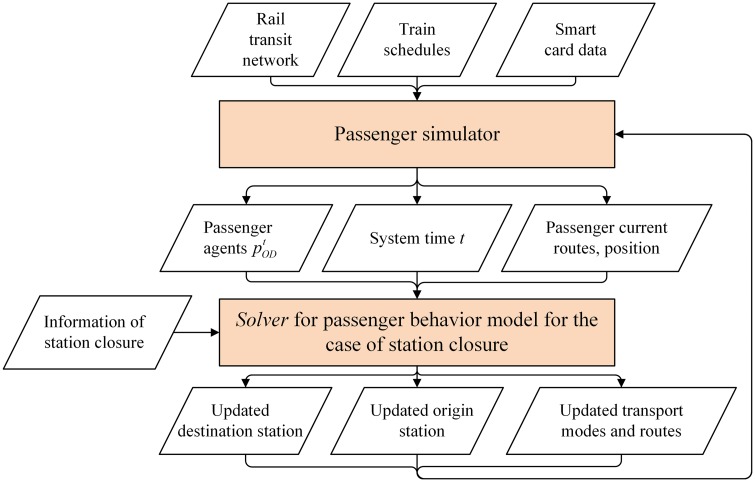
The framework of the integrated solution algorithm. There are two core modules in the solution algorithm: the passenger simulator and the model solver. The main inputs of the passenger simulator are the rail transit network, train schedules, smart card data, and bus/taxi data. In addition, the simulator provides instant passenger information, including the current path, station, and train, for the tens of thousands of behavior optimization models. The closure duration and its overestimation are also the inputs of the model solver. Finally, the outputs of the model solver, namely, the updated destination station, origin station, the transport mode and paths, are computed and then become the inputs of the passenger simulator.

As the decision variables, constraints and some key parameters are time-depended, the passenger behavior optimization model we construct belongs to the category of dynamic 0–1 integer programming problems. Some mathematical algorithms have been developed to solve similar discrete problems [34,35]. But it is still difficult for existing algorithms to solve our proposed model while some dynamic parameters are complex and changeable over time. Therefore, an integrated solution algorithm for solving the model is developed. This algorithm contains two modules: the passenger simulator and the basic model solver. The relationship between the two modules is demonstrated in Fig 4.

The passenger simulator is developed to simulate passenger movements in the rail transit network in real time, mainly based on train schedules and huge amounts of smart card data. In addition, it provides a particular passenger with the planned origin station and destination station, the system time, the current state, the current path and the position of each passenger for our model solver. Furthermore, the simulator also estimates the effect of station closures on passenger demand at the disaggregate level.

The basic model solver is developed to solve the passenger behavior model for the station closure case for an individual passenger at a particular time. The solver outputs the alternative station (replacing the origin station or the destination station), the alternative transport mode for bridging the alternative station and the closed station, and the path of each alternative transport mode. These types of updated passenger travel information are then fed back to the passenger simulator. Changeable station closure information is also required for the model solver.

#### Passenger simulator module

First, some necessary parameters used in the simulation process are introduced. We label *t*_*s*_ as the starting time of the simulation, *t*_*e*_ as the ending time of the simulation, *T*_*d*_ as the duration of each simulation period, Δ*t* as the simulation step, $t_{i}^{p}$ as the entry time of passenger *p* at station *s*_*i*_, $s_{p}^{t}$ as the current location of passenger *p* at time *t*, *t*_*p*_ as the current time of passenger *p*, $d_{i}^{v}$ as the departure time of train *v* at station *s*_*i*_, and $a_{i}^{v}$ as the arrival time of train *v* at station *s*_*i*_. *state*_*p*_ is the state of passenger *p* and can be “In”, “Entry”, “Waiting”, “Transfer”, “Boarding”, “Alighting”, “Exit”, or “Out”.

***“In”***: indicates that the passenger agent has just been generated.

***“Entry”***: indicates that the passenger agent has just completed checking in at entry gates and is going to the platform to wait for a train.

***“Waiting”***: indicates that the passenger agent is waiting on the platform for a train.

***“Boarding”***: indicates that the passenger agent has completed the boarding process and is now on a train.

***“Transfer”***: indicates that the passenger agent has completed the alighting process and is now transferring to another platform or train.

***“Exit”***: indicates that the passenger agent has completed the alighting process and is now going to check out at the exit gates of the destination station.

***“Out”***: indicates that the passenger agent has completed checking out and is now out of the rail transit system.

In addition, some constant parameters concerning the passenger walking time consumption are introduced: *tIn*_*i*_ donates the average walking time at station *s*_*i*_ from the check-in gate to the platform, *tOut*_*i*_ is the average walking time for station *s*_*i*_ from the platform to the check-out gate, and $tr_{j}^{i}$ is the average walking time for transferring from the departure platform of station *s*_*i*_ to the target platform of station *s*_*j*_.

**Fig 5:**
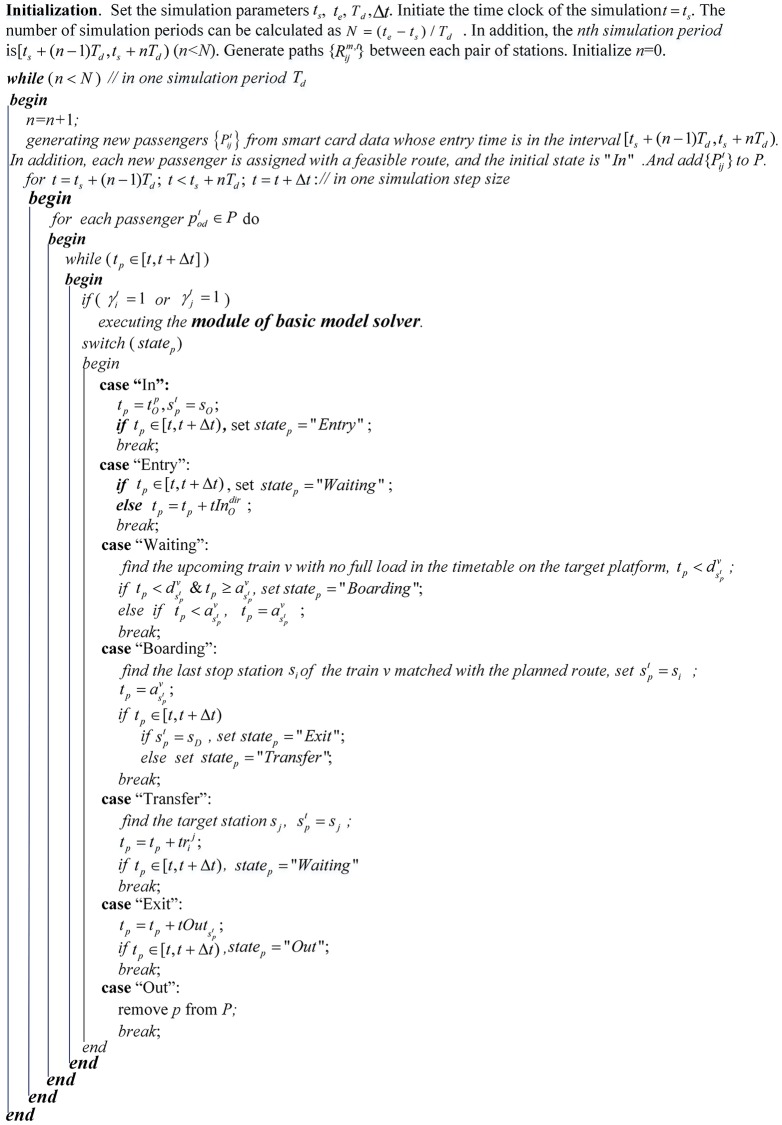
The passenger simulator module. The parameters are initialized firstly. And then the in each simulation step of each simulation period, each passenger will get the most out of what they can do. One passenger's single rail journey will start from the entering a station, and ends up with exiting a station. The whole process will be tracked by the transitions of passenger states, namely “In”, “Entry”, “Waiting”, “Transfer”, “Boarding”, “Alighting”, “Exit”, or “Out”.

Finally, the passenger simulation algorithm is introduced in Fig 5.:

#### Basic model solver module

**Fig 6:**
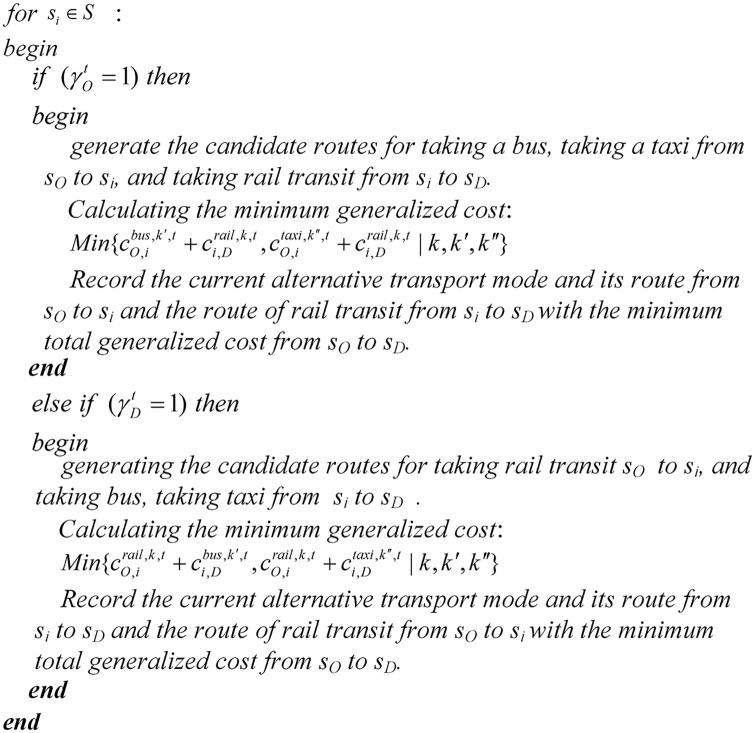
The basic model solver module. For each passenger, all stations will be traversed and examined with the objective of passenger behavior optimization model. And then the station with the minimum model objective will be chosen as the alternative station.

The basic model solver, shown in Fig 6, is embedded in the simulation algorithm in front of the “switch” statement. For a particular passenger, the system time, current state, current path and position of each passenger are provided by the simulator. The outputs of the passenger behavior model are produced after a station closure occurs. Passengers update their current station, planned origin/destination station, current state and current time parameters, which are then fed back to the simulator.

## Case study

### Background

#### Beijing rail transit network

**Fig 7:**
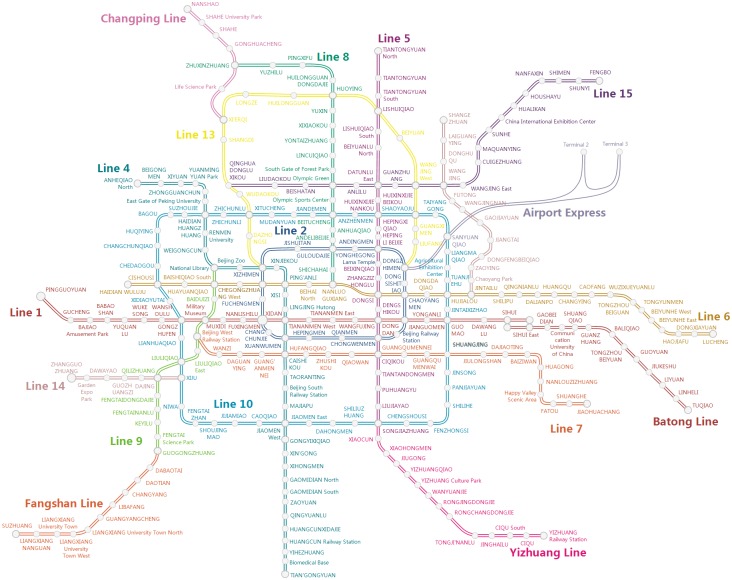
The Beijing rail transit network map for 2015. This map is originally presented in this paper by the ***Haodong Yin***, based on open access data of the Beijing rail transit using our own developed software.

The Beijing rail transit system is one of the busiest tracks in the world. In Beijing, there are more than 10 million riders traveling daily through the heavily utilized rail transit network, which connects 17 lines with a total length of 561 kilometers and 328 operating stations (a single transfer station is split into two stations if two lines join together at the transfer station); see Fig 7. Station closures have become frequent in the Beijing rail transit’s daily operations and has a large impact on passengers’ regular journeys, such as increasing their journey time and disrupting their normal travel plans.

#### Data preparation

Although many parameters are considered in the above behavior optimization models and simulation algorithms, two main types of parameters should be obtained: (1) smart card data used to generate the passenger agents and (2) the time and money consumptions of buses or taxis for trips between each pair of rail stations, which are the basic input parameters of the proposed behavior model. Moreover, the basic rail transit network of the Beijing rail transit, the planned timetables of each rail transit line, the inbound capacity of each station, and the parameters concerning the passenger walking time are also acquired.

**(1) Smart card data of the Beijing rail transit**

The data are used in the passenger simulation. Smart card data can be acquired through the Beijing rail transit’s automatic fare collection (AFC) system and is used to generate the passenger agents. Four essential elements are our focus: the entry rail station, the entry time, the exit rail station and the exit time. Every weekday, nearly 5 million items of valid transaction details of smart cards are processed. In this article, the smart card data from 19/5/2015 from 4 o’clock to 12 o’clock are processed. A few examples of smart card data are listed in Table 1.

**Table 1:** Example of smart card data.

ID	Entry station	Entry time	Exit station	Exit time
**1**	XIDAN	2015/5/19 8:05:24	Beijing Railway Station	2015/5/9 8:18:24
**2**	XIDAN	2015/5/19 5:27:00	DONGZHIMEN	2015/5/19 5:58:20
**3**	QIANMEN	2015/5/19 7:20:30	Beijing Railway Station	2015/5/19 7:31:22
**…**	…	…	…	…

**(2) Bus and taxi cost**

The **bus and taxi cost** data are the key input for the passenger behavior models. ***Direction API*** is adopted to obtain the expected time and money consumptions of buses and taxis between each two rail stations. ***Direction API*** is a free and open access application programming interface developed by ***Baidu***, Inc. It provides services for traveler guidance using buses, driving and walking using *http* based on the ***Baidu*** map in real time and can retrieve data in *xml* or *json* formats. We process the data file with C# programming tools according to the provided data structure on the *Baidu* developer’s main page [31]. Some examples are listed below in Table 2.

**Table 2:** Example of expected fare and time consumption of buses and taxis between each pair of rail stations.

ID	Station O	Station D	Fares of direct bus (RMB)	Time required for the bus (min)	Taxi fares (RMB)	Time required for the taxi (min)
**1**	JISHUITAN	GULOUDAJIE	2	20.8	18	12.4
**2**	JISHUITAN	PING’ANLI	2	17.1	17	9.6
**3**	Beijing Railway Station	JISHUITAN	3	81	33	28.7
**…**	…	…	…	…	…	…

### Case study descriptions

#### Case One: JISHUITAN as the closed station

**Fig 8:**
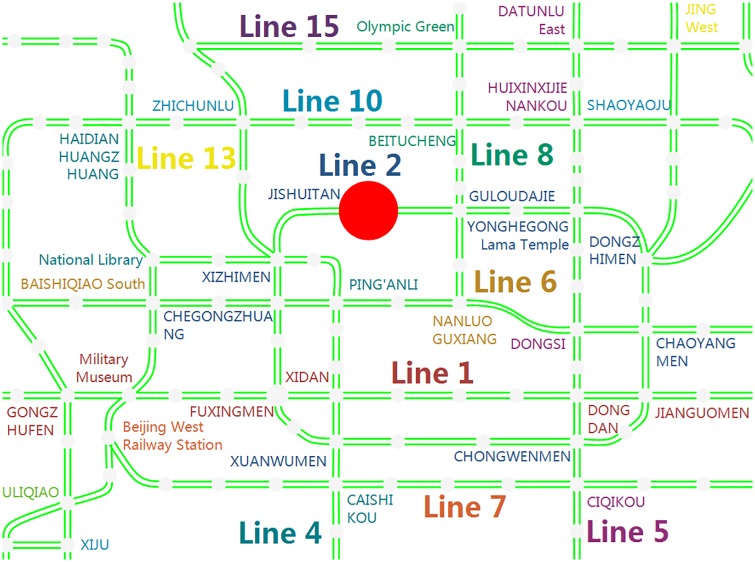
The location of JISHUITAN station in the Beijing rail transit network. The red point is JISHUITAN station.

**Fig 9:**
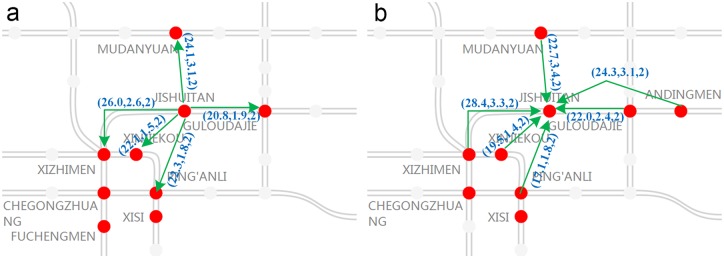
The total time consumption, distance and fee between JISHUITAN station and nearby stations. The parameters (*t*, *d*, *f*) on each arc that are marked by a red arrow are the total journey time (minutes), distance (kilometers) and fee (RMB) by bus, including the walking during the trip. (a) denotes the travel cost from the closed station to some nearby stations. (b) indicates the travel cost from the nearby stations to the closed station. It should be noted that the travel costs on the arcs in the different directions between the pairs of stations are different.

In the first case study, JISHUITAN station is selected as the disrupted station. The duration of the station closure is from 7:30 to 8:30 on Tuesday, May 19, 2015. JISHUITAN station on Line 2 is among the busiest stations during morning rush hour, and the location is shown in Fig 8. Furthermore, to intuitively display the relationship between JISHUITAN station and the nearby stations, the travel cost by bus is shown in Fig 9.

#### Case Two: TIANTONGYUAN as the closed station

**Fig 10:**
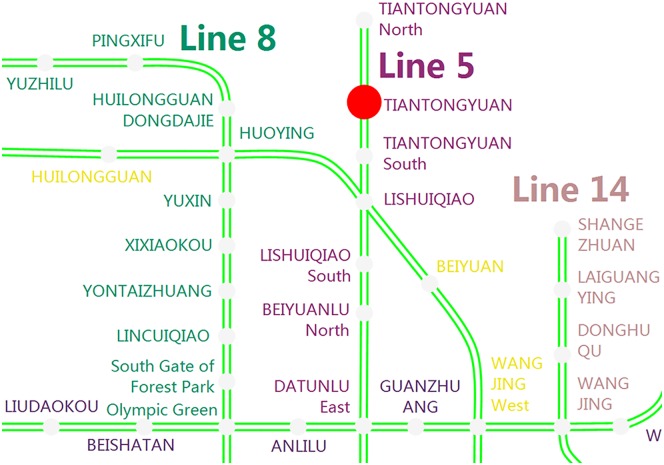
The location of TIANTONGYUAN station in the Beijing rail transit network. The red point is the TIANTONGYUAN station.

**Fig 11:**
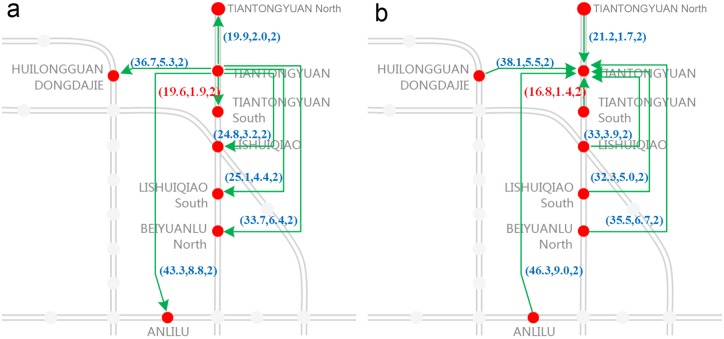
The total time consumption, distance and fee between the TIANTONGYUAN station and nearby stations. The parameters (*t*, *d*, *f*) on each arc that are marked by a red arrow are the total journey time (minutes), distance (kilometers) and fee (RMB) by bus, including the walking during the trip. (a) denotes the travel cost from the closed station to some nearby stations. (b) indicates the travel cost from the nearby stations to the closed station. It should be noted that the travel costs on the bidirectional arcs between the same two stations may be different.

In the second station closure case study, TIANTONGYUAN station is set to be closed. In addition, the duration of the station closure is also from 7:30 to 8:30 on Tuesday, May 19, 2015. TIANTONGYUAN station of Line 5 is located in the Tiantongyuan area, which is among the top large-scale residential areas of Beijing. The location is shown in Fig 10. In addition, the travel cost between the TIANTONGYUAN and the nearby stations by bus is shown in Fig 11.

#### Simulation experiment groups settings

To validate the behavior model under station closure conditions, a set of simulation experiments are designed, as listed in Table 3. The simulation period is set from 4:00 to 12:00. Every station is also set to have an inbound capacity according to the capacity of equipment and facilities according to the historical data.

**Table 3:** The different values of the parameters in the simulation cases.

ID	Cases	g	Closure duration	Simulation period	Description
1	Experimental Group 1: **JISHUITAN** as the closed station	1	[7:30,8:30]	[4:00,12:00]	The 1^st^ Closure Scenario
2		1.5	[7:30,8:30]	[4:00,12:00]	The 2^nd^ Closure Scenario
3		2	[7:30,8:30]	[4:00,12:00]	The 3^rd^ Closure Scenario
4		3	[7:30,8:30]	[4:00,12:00]	The 4^th^ Closure Scenario
5		5	[7:30,8:30]	[4:00,12:00]	The 5^th^ Closure Scenario
6		10	[7:30,8:30]	[4:00,12:00]	The 6^th^ Closure Scenario
7	Experimental Group 2: **TIANTONGYUAN** as the closed station	1	[7:30,8:30]	[4:00,12:00]	The 7^th^ Closure Scenario
8		1.5	[7:30,8:30]	[4:00,12:00]	The 8^th^ Closure Scenario
9		2	[7:30,8:30]	[4:00,12:00]	The 9^th^ Closure Scenario
10		3	[7:30,8:30]	[4:00,12:00]	The 10^th^ Closure Scenario
11		5	[7:30,8:30]	[4:00,12:00]	The 11^th^ Closure Scenario
12		10	[7:30,8:30]	[4:00,12:00]	The 12^th^ Closure Scenario
13	No-treatment Control Group	~	~	[4:00,12:00]	Normal situation with no closures

The overestimation of the closure duration is comprehensively investigated by adopting different values of the parameter *g*. In addition, the effects of station closures on passengers’ individual behavior and passenger demand can be estimated for different *g*. We use the time value *α*^*m*^ of 25 according to previous studies on Chinese cities [32, 33].

### Results and discussion

To find the solutions of the passenger behavior model for the case of a station closure, our behavior model and the algorithm are implemented with C# and are performed on a PC with a 3.2 GHz, Intel(R) Core(TM) i5-3470 processor and 16 GB of RAM running Windows 7 (64 bit). The object-oriented programming technology, which is the mainstream programming technology, is adopted in our implementation.

In the following discussions, the general introduction of the simulation results is firstly given, where huge data and its display are shown. And then, the dynamic effects of closure duration and its overestimation on passenger behavioral choices and passenger demand are presented. Finally, a questionnaire survey is conducted and the results are compared with the model outputs.

#### General introduction of the simulation results

**Fig 12:**
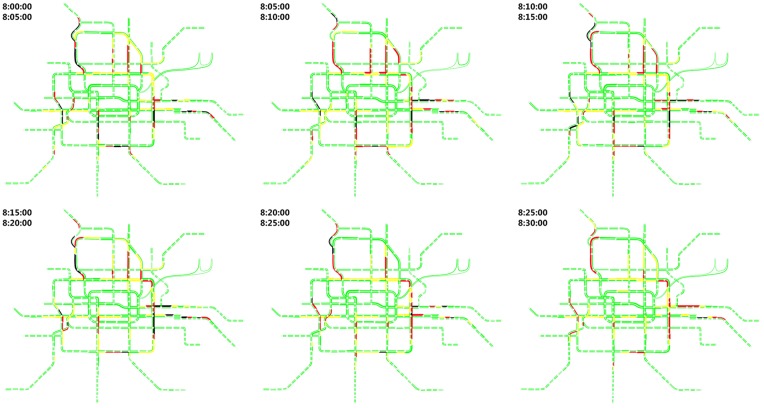
The passenger flow status at every 5 minutes on the rail network from 8:00–8:30. We can simulate every moment of each passenger in our passenger simulator. In addition, once trains enter the segment, the passengers in the trains is counted by the segment, and the train’s capacity is also merged. If ***N*** stands for the total passenger count and ***Ca*** stands for the capacity of all the trains passing through the segment, the train load of the segment can be calculated as ***N***/***Ca***. We use four colors to display the train load of each segment: lime indicates a load range from zero to 80%; yellow indicates a load from 80% to 100%; red indicates a load from 100% to 120%; and black indicates that the load is greater than 120%.

**Fig 13:**
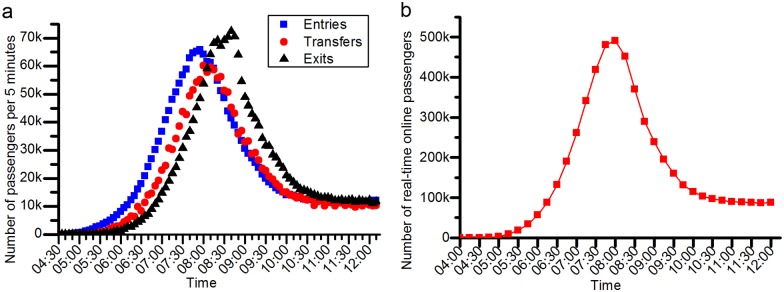
Online passengers in the rail transit network. (a) represents the total entries, transfers and exits of the rail transit network at every 5 minutes. (b) shows the number of instantaneous passengers on the rail transit network at the end of each simulation period. The number of passengers reaches its peak, namely, 490,397, at end of the simulation period [8:15, 8:30). The simulation results are computed in the normal situation with no station closure.

**Fig 14:**
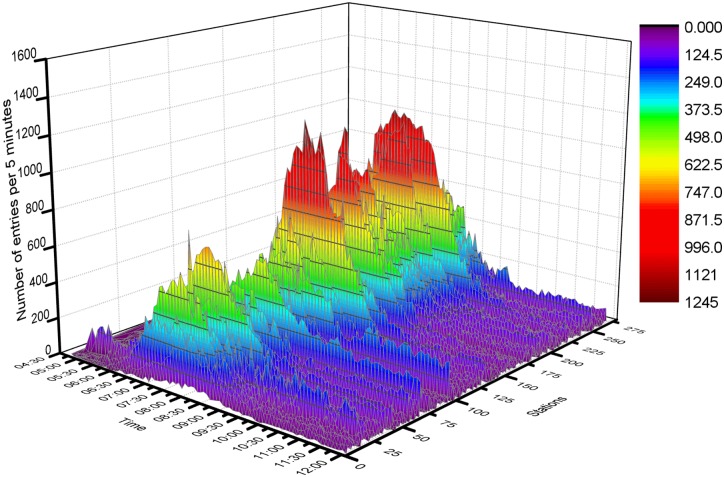
Number of entries in each station every 5 minutes. This graph has three dimensions: time, stations and the number of passengers. Every point in a colored area donates the entries of a particular station during a particular period. The color indicates the number of passengers entering the station.

**(1) Hug data and the simulation results**. Approximately 2,074,267 items of smart card data from 4 o’clock to 12 o’clock are processed in our developed simulator. The simulation results are displayed in Fig 12. We divide the whole simulation process into 32 periods. Fig 13(a) shows the total dynamical entries, transfers and exits in the whole rail transit network in each simulation period. The number of instantaneous online passengers on the rail transit network at the end of each simulation period is also shown in Fig 13(b). The maximum number of instantaneous online passengers reaches nearly 500,000. The entries in hundreds of stations during the 32 simulation periods are shown intuitively and dynamically in Fig 14.

**Fig 15:**
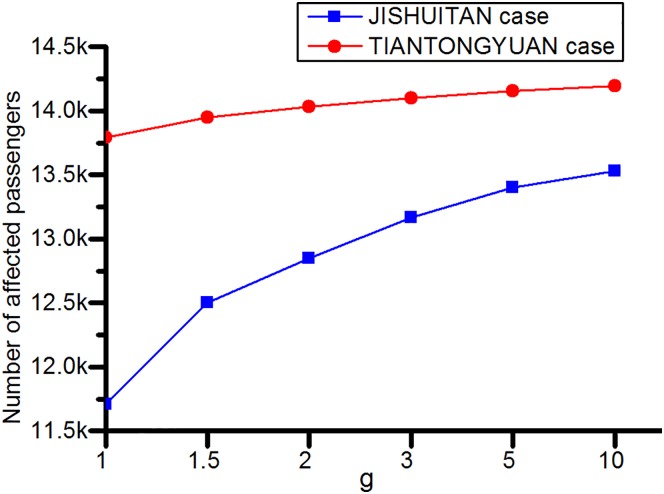
Affected passengers for varying overestimation of the closure duration. A longer announced closure duration, that is, a larger overestimation of the closure duration, results in more affected passengers.

**(2) Total affected passengers**. We define the affected passengers as those who have to wait for extra time for the recovery of the closed station, alter their departure or destination stations, or give up their rail transit journey. As the announced closure duration increases, more passengers are affected, as shown in Fig 15. In other words, if the rail transit operator can accurately estimate the closure duration, fewer passengers will be affected.

#### Effects of a station closure on passenger behavioral choices

**Fig 16:**
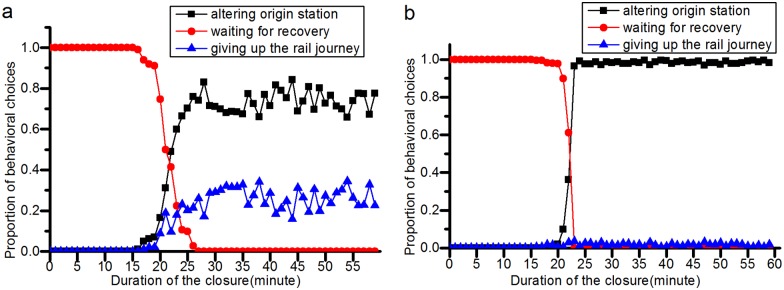
The relationship between the closure duration and the passenger behavioral choices. (a) represents the simulation results in the JISHUITAN case. (b) denotes the simulation results in the TIANTONGYUAN case.

**Fig 17:**
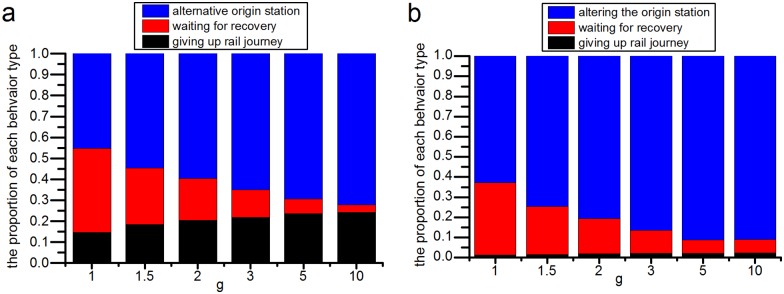
The relationship between the overestimation of the closure duration and the passenger behavioral choices. (a) shows the proportion of passenger behavioral choices after the station closure in the JISHUITAN case. (b) shows the proportion of passenger behavioral choices after the TIANTONGYUAN station is closed in the second closure case. With increasing *g*, the number of passengers taking an alternative origin station or giving up on their rail transit journey gradually increases.

**(1) Passengers’ behavior choices will change as the closure duration or its overestimation changes**. Fig 16 shows that with increasing closure duration, more passengers will alter their planned origin stations or quit their rail transit journey, and fewer passengers will wait at the closed station for its recovery. A similar conclusion can be drawn when *g* increases, as shown in Fig 17. With increasing *g*, which means that the rail transit operator’s overestimation of the closure duration increases, the number of passengers who alter their planned origin stations or give up on their rail transit journey gradually increases, and the number of passengers waiting at the closed station decreases.

**Fig 18:**
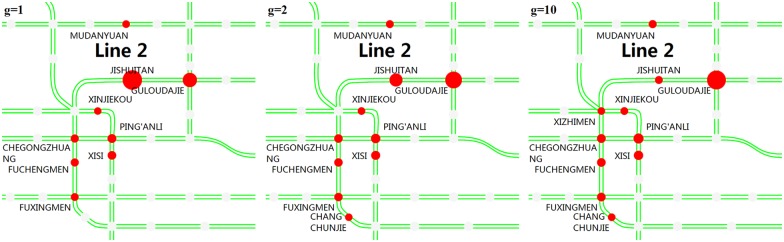
The main stations that passengers choose as their alternative origin stations after JISHUITAN station is closed. The red circles represent the alternative origin stations, including the closed JISHUITAN station. The size of each circle represents the number of passengers who choose that station, and a larger radius indicates relatively more passengers.

**Fig 19:**
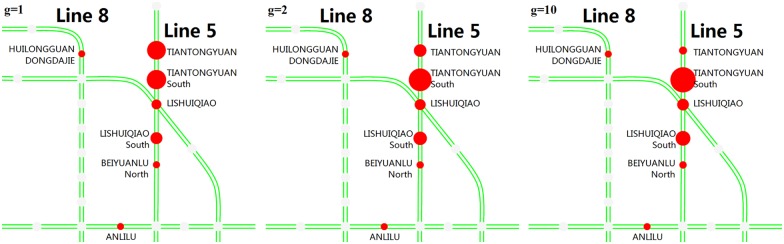
The main stations that passengers choose as their alternative origin stations after TIANTONGYUAN station is closed. The red circles represent the alternative origin stations. The size of each circle represents the number of passengers who choose that station, and a larger radius indicates relatively more passengers.

**(2) Alternatives of origin stations**. Figs 18 and 19 show in detail the top stations that passengers prefer to choose as their alternative origins when their planned origin stations are closed. Fig 18 shows the main stations that passengers choose as their alternative origin stations after JISHUITAN station is closed. We can see that GULOUDAJIE station to the east of the closed station always attracts the most passengers. Fig 19 shows the main stations that the passengers choose as their alternative origin stations after TIANTONGYUAN station is closed. The TIANTONGYUAN South station ranks first in attracting passengers from the closed station. It can also be seen that a larger overestimation of the closure duration, that is, a larger *g*, will result in fewer passengers waiting at the closed station and more passengers altering their planned origin station.

**Fig 20:**
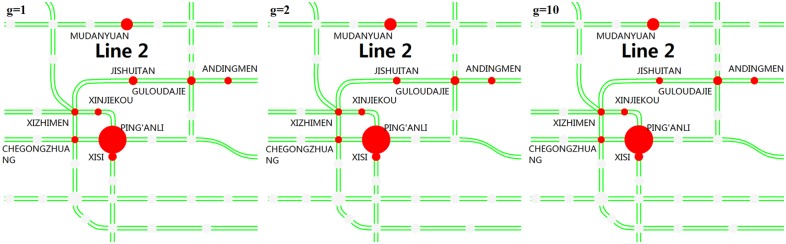
The main stations that passengers choose as their alternative destination stations after JISHUITAN station is closed. The red circles represent the alternative destination stations, and a larger radius indicates a larger proportion.

**Fig 21:**
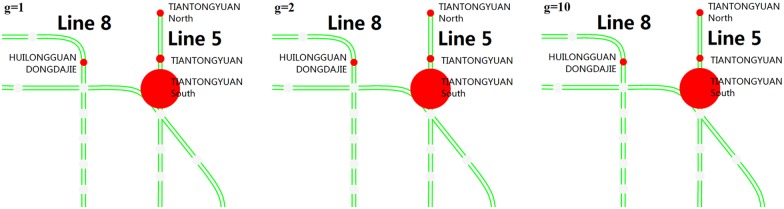
The main stations that passengers choose as their alternative destination stations after TIANTONGYUAN station is closed. The red circles represent the alternative destination stations, and a larger radius indicates a larger proportion.

**(3) Alternative destination stations**. Figs 20 and 21 show the top stations that passengers choose as their alternative destinations when their planned destination stations are closed. PING’ANLI station is the most selected station after the JISHUITAN station is closed, and TIANTONGYUAN South is the most selected station after the TIANTONGYUAN station is closed. Passengers’ choices of alternative destinations are relatively more concentrated than the choices of alternative origins.

#### Effects of the station closure on the passenger flow demand

**Fig 22:**
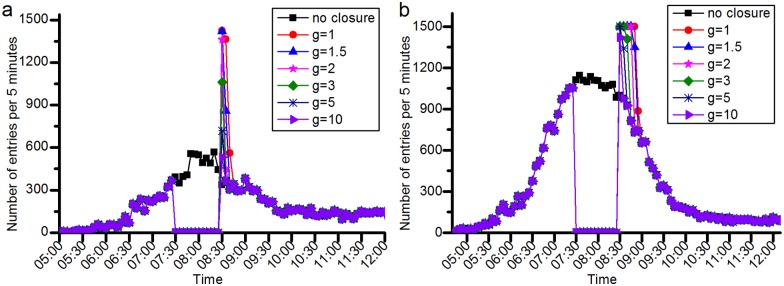
The number of entries at the closed stations for varying values of *g*. (a) shows the number of entries at JISHUITAN station. (b) represents the number of entries at **TIANTONGYUAN** station. As shown in the first closure case in Fig 22(a), when g equals 1, JISHUITAN station experiences 2343 more entries than in the no closure situation after 8:30. However, in the case of g equal to 10, this difference is 197, which is 91.7% smaller than in the case of g equal to 1. The duration is 75 minutes in the case of g equal to 10, which is 16.7% more than in the case of g equal to 1. As shown in the second closure case in Fig 22(b), when g equals 1, the TIANTONGYUAN station experiences 3195 more entries than in the no closure situation after 8:30. However, in the case of g equal to 10, this difference is 422, which is 86.8% less than in the case of g equal to 1. The duration is 90 minutes in the case of g equal to 10, which is 38.5% more than the duration in the case of g equal to 1.

**(1) Changes in the passenger flow at the closed stations**. Fig 22 shows the effects of the station closure on the passenger flow at the closed station. More passengers choose to wait at the planned origin station for station recovery when the overestimation of the closure duration is smaller. When the closure ends, the waiting passengers gradually begin to enter the closed station, which leads to more entries than normal. Moreover, every station has a limited inbound capacity (the inbound capacity of JISHUITAN is 1362 persons per 5 minutes, and TIANTONGYUAN is 1500 persons per 5 minutes). Therefore, a certain number of passengers in some closed stations may need to wait for a longer time due to the limited inbound capacity. As a result, it takes longer than the closure duration itself for the entries of some closed stations to return to normal levels, as shown in Fig 22(b). Fig 22 also indicates that the duration of the closure’s influence on some closed stations will increase with decreasing *g*.

**Fig 23:**
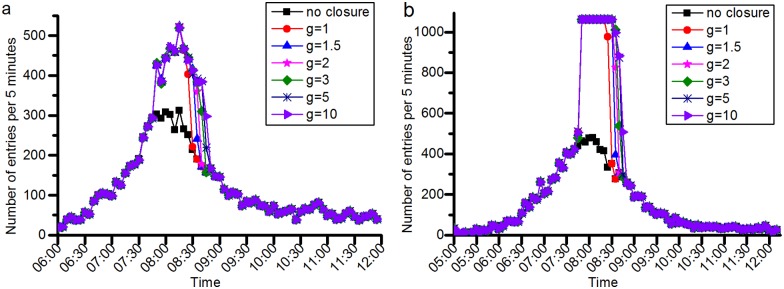
The number of entries at the affected stations for varying values of *g*. (a) shows the number of entries at GULOUDAJIE station in the JISHUITAN case. (b) shows the number of entries at TIANTONGYUAN South station in the TIANTONGYUAN case. The inbound capacity of the GULOUDAJIE is 1000 persons per 5 minutes, and the TIANTONGYUAN South station is 1062 persons per 5 minutes. As shown in the first closure case in Fig 23(a), when g equals 1, the GULOUDAJIE station experiences 1,284 more entries than in the no closure case. However, in the case of g equal to 10, this difference is 2,064, which is 60.9% more than that in the case of g equal to 1. The duration in the case of g equal to 10 is 60 minutes, which is 33.3% more than in the case of g equal to 1. As shown in the second closure case in Fig 23(b), when g equals 1, the TIANTONGYUAN South station experiences 4,979 entries more than in the no closure case. However, in the case of g equal to 10, this difference is 7294, which is 46.5% more than that in the case of g equal to 1. The duration in the case of g equal to 10 is 65 minutes, which is 44.4% more than in the case of g equal to 1.

**(2) Changes in the passenger flow at the nearby stations**. Fig 23 shows the effects of the station closure on the passenger flow at stations near the closed stations. The closure of JISHUITAN station results in a significant increase in the number of entries at the nearby stations. With increasing *g*, the nearby stations suffer a more significant impact due to the closure, and the severity and duration of the closure impact increase greatly.

#### Comparisons of model results and manual survey

**Fig 24:**
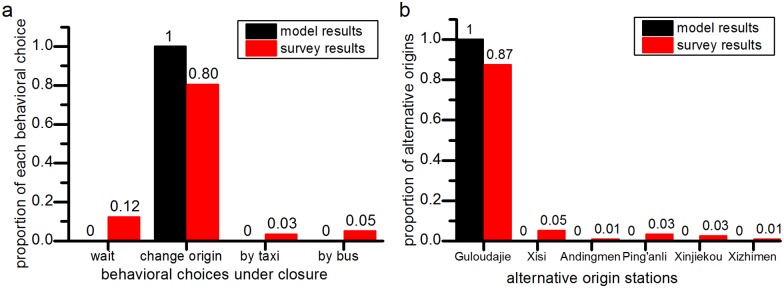
The comparisons of the model outputs and the manual surveys in the JISHUITAN closure case. In the manual survey, 80% (119 of 148) of passengers choose to alter their planned origin station (Fig 24(a)) and 87% (104 among the 119) passengers choose the GULOUDAJIE as their new origin station (Fig 24(b)). But our model results show that all the affected passengers will change their origins and choose the GULOUDAJIE station as their new origins, which indicates that there is an error of no more than 20% between the model results and the actual survey results.

To validate our behavior model, we conduct a questionnaire (S1 File) about the passengers’ choices about alternative origin stations when their planned origin station is closed. One pair of OD from the **JISHUITAN** station of Line 2 to the **DAWANGLU** station of Line 1 is selected to create a station closure scenario with a closure duration of 30 minutes and *g* equal to 1. Finally, 149 answers are collected. The model results and the manual survey data are shown in Fig 24. The comparisons of the model outputs and the manual survey show that the accuracy of our proposed behavior optimization model is approximately 80%.

## Conclusions

We develop a novel approach for estimating the impact of alternative station closure scenarios in a rail transit network on passenger behavioral choices at the individual level and on passenger demand at the disaggregate level. An agent-based passenger behavior optimization model for a station closure is proposed for the first time. To solve the model, an integrated solution algorithm with a passenger simulator is developed in this article. To address the passenger’s uncertainty of the station closure duration, a parameter indicating the overestimation of the closure duration is adopted in our proposed model. The Beijing rail transit system is introduced as a case study using real-world smart card data and online urban transit data. The JISHUITAN station and the TIANTONGYUAN station are tested as the closed stations in our case studies. The effects of the closure duration and its overestimation on passenger behavioral choices and passenger flow demand are discussed. The following conclusions can be drawn:

1. Our proposed behavior optimization model is valid for capturing passenger behavior during a station closure at an approximate accuracy of 80%, which is supported by the results of a manual survey. Moreover, our developed model and simulation-based algorithm can dynamically provide quantitative results about the effect on passenger demand.
2. When a station closure happens, the affected passengers prefer choosing alternative stations with convenient transitions from the closed station to the new origin station or from the new destination station to the closed station. In addition, with increasing closure duration or its overestimation, more passengers will alter their planned origin stations or give up on their rail transit journey, and fewer passengers will wait at the closed stations for the station recovery.
3. It may take longer than the closure duration itself for the entries of the closed station, with limited inbound capacity, to return to normal levels. Moreover, with increasing *g*, the duration of the closure’s influence on the closed stations will decrease. However, the nearby stations suffer a more significant impact due to the closure, and the severity and the duration of the closure impact increase greatly.
4. Furthermore, if the rail transit operator can more accurately estimate the closure duration (namely, as *g* approaches 1), the impact of the closure can be somewhat mitigated.

In our future research, the effect of alternative station closure scenarios, including different closure durations and their overestimation, on the passenger journey time will be investigated. Passenger behavior models with different granularities will be further developed and validated.

## Supporting Information

S1 File A questionnaire on the passenger departure station selection behavior under a station closure. This is a questionnaire on the passenger behavior under a station closure. The closure durations are set as 15 minutes, 30minutes and 60 minutes respectively.
(DOC)
