# Supplementary material for: Modeling and Simulating Passenger Behavior for a Station Closure in a Rail Transit Network
Source: PLoS One. 2016 Dec 9;11(12):e0167126. doi: 10.1371/journal.pone.0167126 (PMC5147864; doi:10.1371/journal.pone.0167126)
Supplement: S1 File — This is a questionnaire on the passenger behavior under a station closure. The closure durations are set as 15 minutes, 30minutes and 60 minutes respectively. (DOC) [file pone.0167126.s001.doc]

## Supporting information

**S1 File:** **A questionnaire on the passenger departure station selection behavior under a station closure**. This is a questionnaire on the passenger behavior under a station closure. The closure durations are set as 15 minutes, 30minutes and 60 minutes respectively.

# A Questionnaire on the Passenger Departure Station Selection Behavior under a Temporary Station Closure

**Introduction**. In order to understand the effect of the unexpected station closure on passenger behavior, and to support emergency response to the subway emergency for operator, the survey is conducted. There are 10 questions in total. Firstly, we thank you for taking the time to give strong support! We assume that you know the Beijing subway or local line network in the questionnaire. And, you can query the Baidu map or other maps to get the necessary guidance information. In addition, the station closure refers to the situation that passengers cannot choose the closed station as their departure stations or as the destination stations for some time, and the trains pass through the closed station with no stop.

**(A) The preferences of behavioral choice in the temporary closure station conditions**

**Q1: Assuming you plan to take the subway from 7:30 am departing from JISHUITAN station to DANGWANGLU station, the whole journey costs about 29 minutes. When you arrives at the JISHUITAN subway station and find that it has been temporarily closed for some reason, but was informed about 15 minutes later the station will be recovered. At this point, your choice is:**

○ Continue to wait (is about 41 minutes, subway fare 5 yuan) (Please skip to Q3)


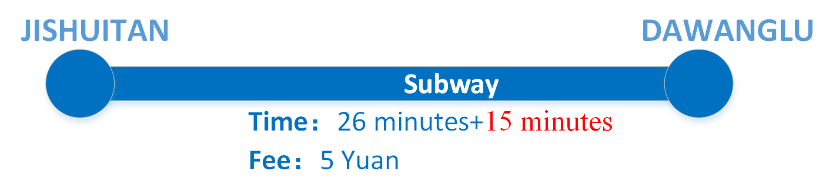


○ Take the bus to the nearby subway station, and then take the subway to reach the destination (the shortest time-consuming about 42 minutes, 6 million)


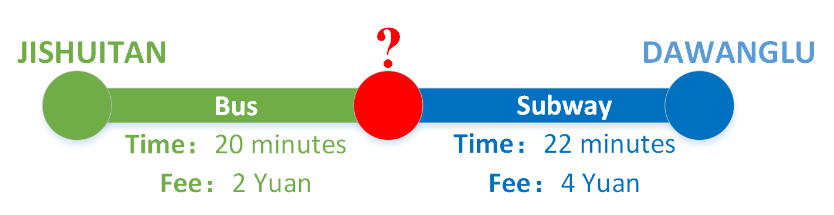


○ Take a taxi to reach the destination (about 42 minutes, playing fee 42 yuan, does not include playing waiting time) (Please skip to Q3)


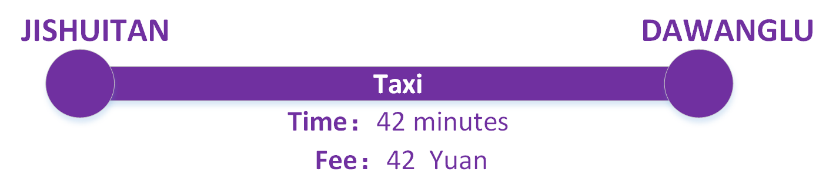


○ Take the bus to reach the destination (about 70 minutes, bus fare 3 yuan, excluding waiting for bus time) (Please skip to Q3)


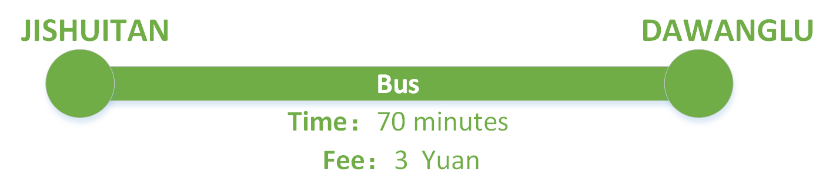


**Q2:** **Now you choose to take the bus to reach the nearby subway station, and then take the subway, then which station will you choose as your alternative departure station ?**

○ GULOUDAJIE station (about 42 minutes, 6 yuan)


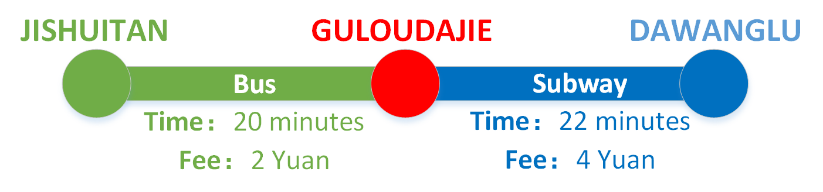


○ XISI station(about 44 minutes, 6 yuan)


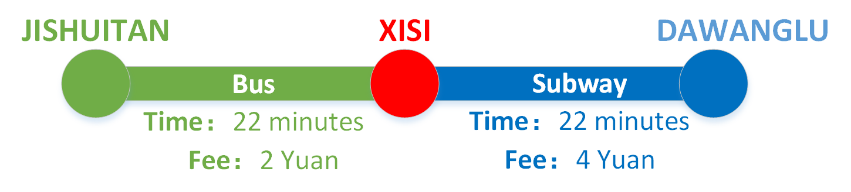


○ ANDINGMEN station (about 47 minutes, 6 yuan)


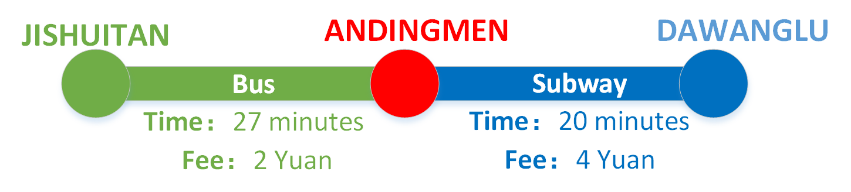


○ PING’ANLI station(approximately 47 minutes, 6 yuan)


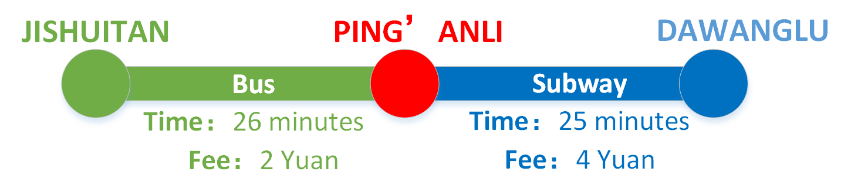


○ XINJIEKOU station (about 49 minutes, 7 yuan)


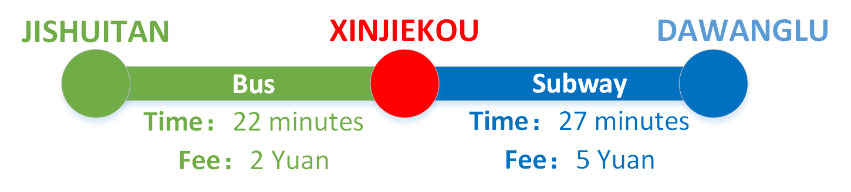


○ XIZHIMEN station (about 54 minutes, 7 yuan)


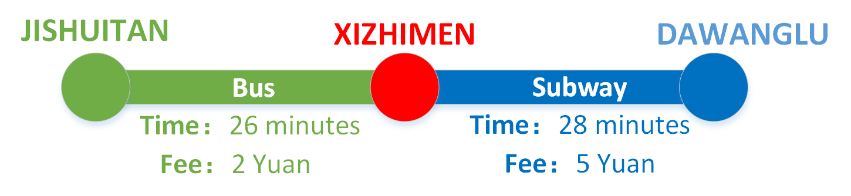


**Q3: If you arrive at JISHUITAN Station and find that JISHUITAN Station is temporarily closed for some reason, and you are told that the station will be in service after about 30 minutes, what are your options?**

○ Continue to wait (about 56 minutes, 5 yuan) (Please skip to Q5)


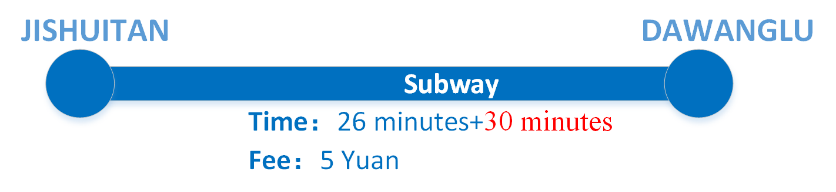


○ Take the bus to the nearby subway station, and then take the subway to reach the destination (the shortest time-consuming is about 42 minutes, 6 million)


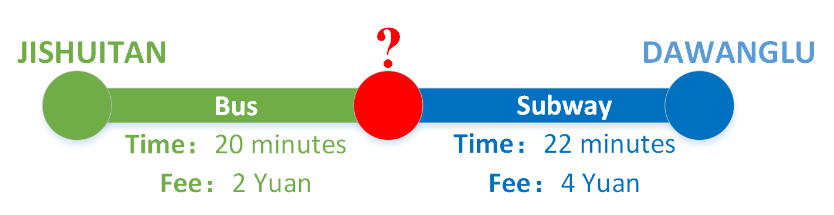


○ Take a taxi to reach the destination (about 42 minutes, playing fee 42 yuan, does not include playing waiting time) (Please skip to Q5)


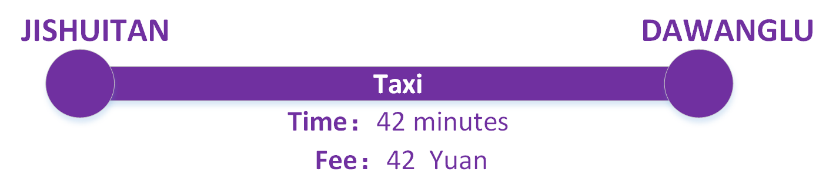


○ Take the bus to reach the destination (about 70 minutes, bus fare 3 yuan, excluding waiting for bus time) (Please skip to Q5)


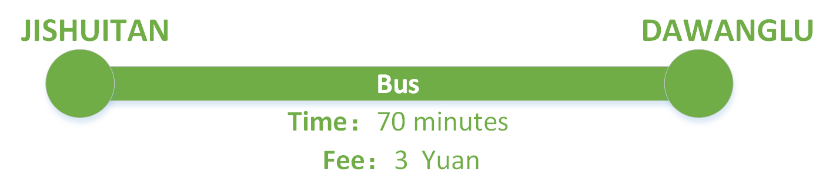


**Q4:** **Now you choose to take the bus to reach the nearby subway station, and then take the subway, then which station will you choose as your alternative departure station ?**

○ GULOUDAJIE station (about 42 minutes, 6 yuan)


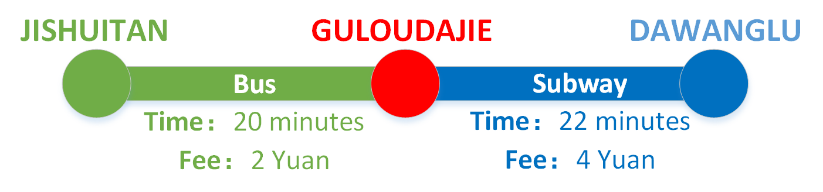


○ XISI station(about 44 minutes, 6 yuan)


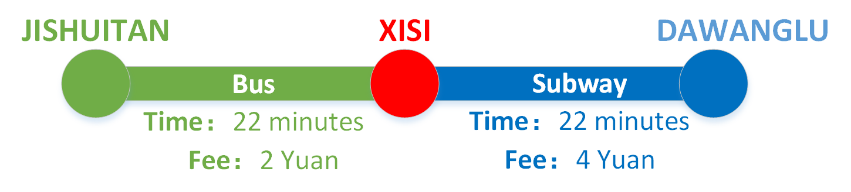


○ ANDINGMEN station (about 47 minutes, 6 yuan)


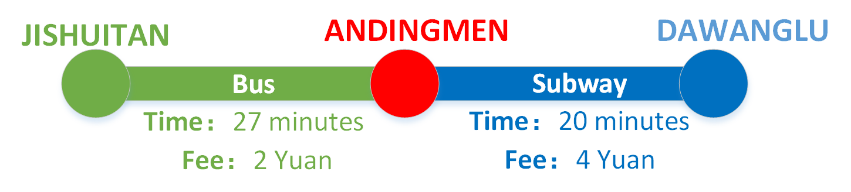


○ PING’ANLI station(approximately 47 minutes, 6 yuan)


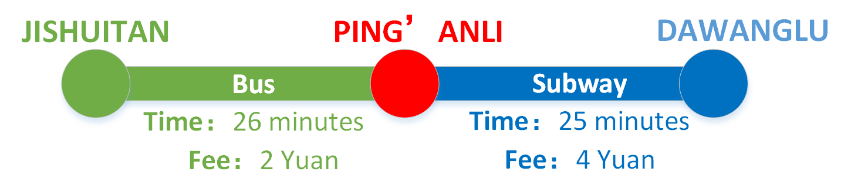


○ XINJIEKOU station (about 49 minutes, 7 yuan)


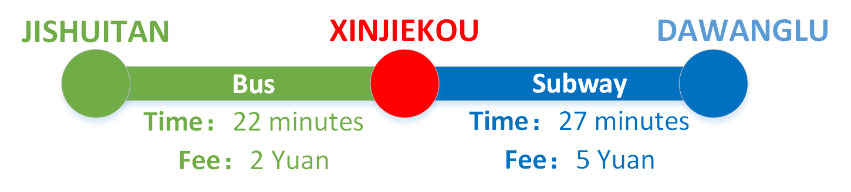


○ XIZHIMEN station (about 54 minutes, 7 yuan)


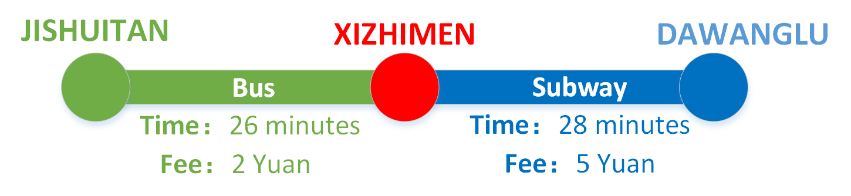


**Q5: If you arrive at JISHUITAN Station and find that JISHUITAN Station is temporarily closed for some reason, and you are told that the station will be in service after about 60 minutes, what are your options?**

○ Continue to wait (about 86 minutes, 5 yuan) (Please skip to Q7)


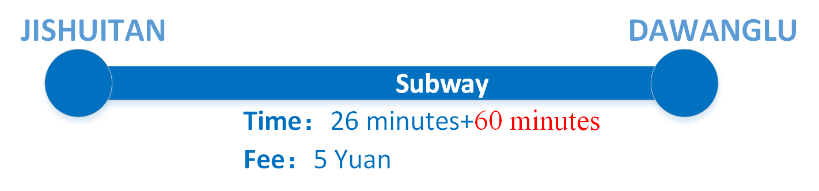


○ Take the bus to the nearby subway station, and then take the subway to reach the destination (the shortest time-consuming is about 42 minutes, 6 million)


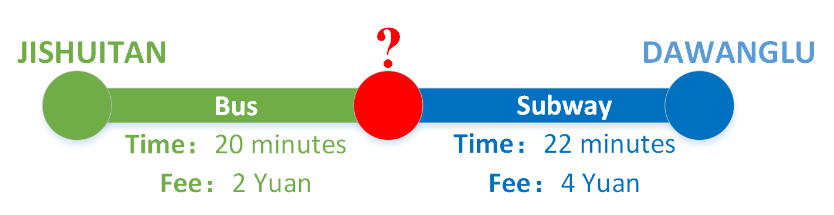


○ Take a taxi to reach the destination (about 42 minutes, playing fee 42 yuan, does not include playing waiting time) (Please skip to Q7)


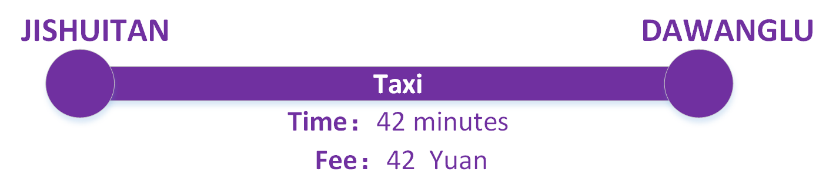


○ Take the bus to reach the destination (about 70 minutes, bus fare 3 yuan, excluding waiting for bus time) (Please skip to Q7)


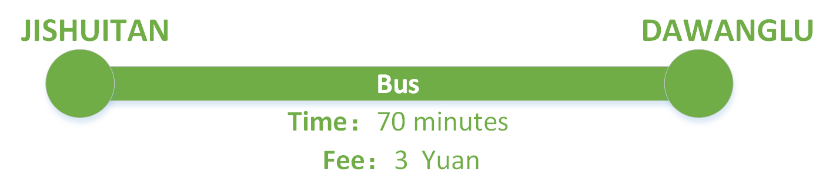


**Q6:** **Now you choose to take the bus to reach the nearby subway station, and then take the subway, then which station will you choose as your alternative departure station ?**

○ GULOUDAJIE station (about 42 minutes, 6 yuan)


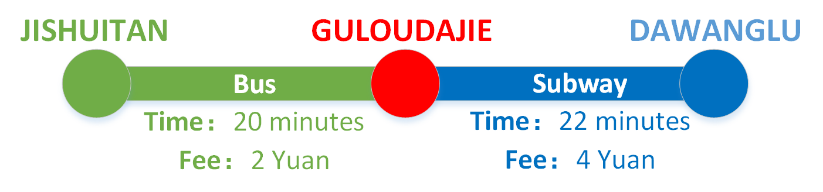


○ XISI station(about 44 minutes, 6 yuan)


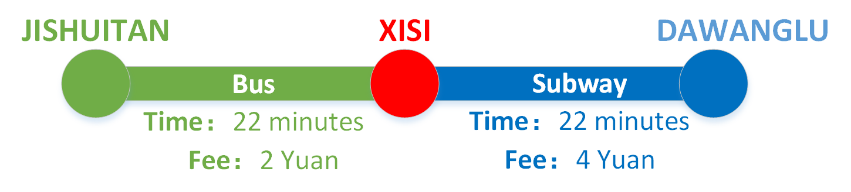


○ ANDINGMEN station (about 47 minutes, 6 yuan)


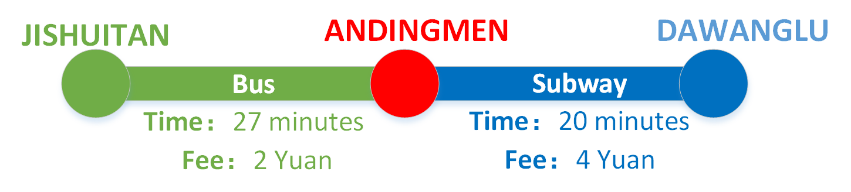


○ PING’ANLI station(approximately 47 minutes, 6 yuan)


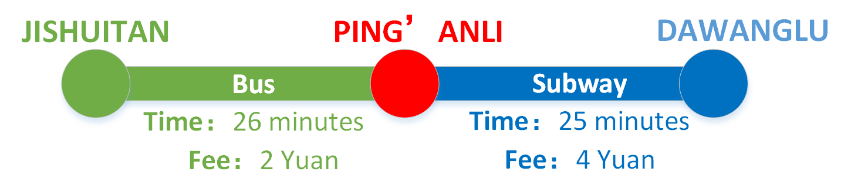


○ XINJIEKOU station (about 49 minutes, 7 yuan)


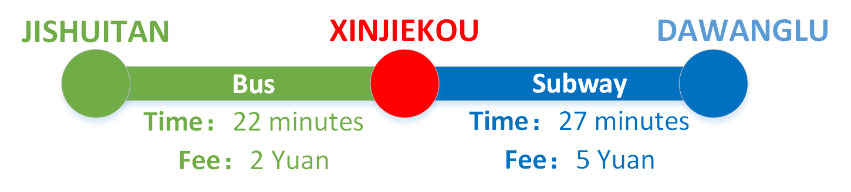


○ XIZHIMEN station (about 54 minutes, 7 yuan)


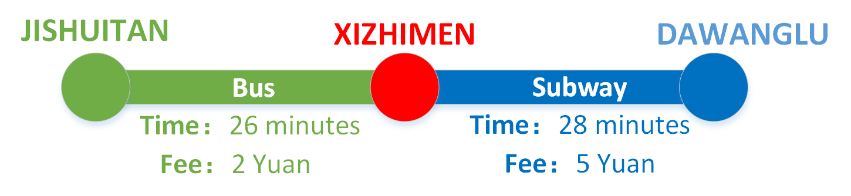


**(B) personal information**

**Q7:** Gender

    ○ Male

    ○ Female

**Q8:** Age

○ Under 18 years old

○ 18 to 25

○ 26 to 30

○ 31 to 40

○ 41 to 50

○ 51 to 60

○ 60 or more

**Q9:** Usually the purpose of your rail transit travel

○ Work

○ Go to school

○ Business

○ visit relatives

○ Others
